# The Relationship between Fundamental Motor Skill Proficiency and Participation in Organized Sports and Active Recreation in Middle Childhood

**DOI:** 10.3390/sports5020043

**Published:** 2017-06-18

**Authors:** Stephanie C. Field, Viviene A. Temple

**Affiliations:** School of Exercise Science, Physical and Health Education, University of Victoria, Victoria, BC V8P5C2, Canada; vtemple@uvic.ca

**Keywords:** physical activity, motor skills, participation, middle childhood, sports

## Abstract

Motor skill proficiency in middle childhood is associated with higher physical activity levels at that age and is predictive of adolescent physical activity levels. Much of the previous research in this area has used accelerometry in determining these relationships, and as a result, little is known about what physical activities the children are engaging in. Therefore the aim of this study was to examine rates of participation in physical activities, the relationships between motor proficiency and how often children participate, and if there were gender-based differences in participation, motor skills, or the relationship between these variables. Participants were 400 boys and girls (Mean age = 9 years 6 months) in grade 4. Motor skills were assessed using the Test of Gross Motor Development-2 (TGMD-2) and physical activity participation was measured using the Children’s Assessment of Participation and Enjoyment (CAPE). Descriptive statistics, chi-squared analyses, and multivariate analysis of variance (MANOVA) were used to examine activity patterns and whether these patterns differed by gender. Correlation coefficients were used to estimate the relationships between fundamental motor skill proficiency and participation. The boys and girls participated in many of the same activities, but girls were more likely to participate in most of the informal physical activities. More boys than girls participated in team sports, boys participated more frequently in team sports, and the boys’ object control and locomotor skill proficiency were significantly associated with participation in team sports. There were some significant associations between motor skills and participation in specific activities; however it is not clear if participation is developing skillfulness or those who are more skilled are engaging and persisting with particular activities.

## 1. Introduction

Motor skill proficiency measured in middle childhood is associated with higher physical activity levels in middle childhood [1] and predictive of adolescent physical activity levels [2]. However, the type of physical activities that are associated with motor skill proficiency in middle childhood is unclear. Participation in physical activities has many benefits for children, physically, socially, and emotionally. Participating in physical activities not only reduces risks of heart disease, diabetes, and obesity [3] but also promotes healthy bone development [4]. Many children who participate in physical activity develop positive self-esteem, increase social interaction, and experience fewer depressive symptoms [5]. Additionally, children who participate in organized physical activities (e.g., team sports) can learn emotional control, conflict resolution, and skills for developing relationships with coaches, friends, and teammates [5].

Boys and girls exhibit different patterns of recreation and leisure participation. There is some evidence that boys tend to participate in more active recreation pursuits such as team sports [6] while girls are more likely to participate in social and skill-based activities (such as hanging out with friends and reading) [7,8,9]. Boys and girls also demonstrate different patterns of motor skill competence. Girls demonstrate stronger locomotor skills (e.g., jumping, hopping, skipping) than boys; while boys demonstrate stronger object control skills (e.g., kicking, catching, throwing) than girls [10,11]. For example, 60% of boys throw proficiently at 5½ years of age, whereas among girls this same proportion (60% throwing proficiently) isn’t seen until 8½ years of age [12]. There is not as much of a gender-based difference in the maturity of other motor skills, although there are differences in when different skills tend to be mature. Sixty percent of boys and girls are proficient in: running at 4–5 years, catching at 6–7 years, hopping at 7 years, and jumping at 10 years of age [12]. While it appears there are some gender-based differences in physical activities, there are some physical activities that show little or no gender based differences such as playing on playground equipment or swimming [6,13].

There is evidence of a positive relationship between motor proficiency, particularly between object control proficiency and accelerometer measured physical activity [1,14,15]; however, there is less evidence of relationships between type of physical activities and motor proficiency. Two related studies, one study with children in kindergarten [13] and one with grade 2 students [16], using the same tools (TGMD-2 and CAPE) and students from the same schools, suggest that the relationship between motor skill proficiency and participation in physical activities may differ for boys and girls as they age. In kindergarten, Temple and colleagues [13] found that boys’ object control proficiency was modestly, but significantly, positively associated with participation in organized sports and locomotor skills were modestly positively associated with participation in active physical recreation. These relationships were very similar for the boys in grade 2, with the addition of a significant positive relationship between the boys’ object control skills and participation in active physical recreation [16]. For girls, however, there were no significant relationships between object control skills or locomotor skills and any sport or recreation categories in kindergarten [13]. In grade 2 there was a weak yet significant positive relationship between girls’ object control skills and participation in active physical recreation, but locomotor skill proficiency was not related to any physical activities, and neither object control skills or locomotor skills were associated with the frequency of girls’ participation in organized sports [16]. The evolution of these relationships for boys and girls in middle childhood has not been examined.

A better understanding of the relationship between motor skill proficiency and recreation and leisure activity participation in middle childhood may help to clarify what is driving participation in physical activity at this age. It would be expected that the relationships between motor skill proficiency and physical activity participation would strengthen as children age as it has been suggested that, in middle childhood, motor competence provides a foundation for participating in sports and physical activities [17]. Therefore, the primary aim of this study was to examine associations between fundamental motor skill proficiency and active physical recreation and organized sport participation of boys and girls in grade 4. The secondary aim is to examine gender-based differences in motor skill proficiency and participation in activities.

## 2. Materials and Methods

A cross-sectional design was used to examine the relationships between motor skill proficiency and recreation and leisure activity participation of boys and girls in grade 4. The University of Victoria Human Research Ethics Board as well as the participating school district granted approval for this study.

Children were eligible to participate if they were attending one of eight consented public elementary schools in British Columbia, Canada. Statistics Canada census data indicates that as of 2014, families in Victoria, British Columbia has a median family incomes approximately 10% higher than the national median [18]. In this particular school district, rates of vulnerability (as measured by the Early Development Index) are lower than, or equivalent to other British Columbia school districts [13,19]. Specific rates of vulnerability are: Physical Health and Well-Being, 13% versus 13.5%; Social Competence, 15% versus 14.5%; Emotional Maturity, 13% versus 13.8%; Language and Cognitive Development, 9% versus 10.3%; and Communication Skills, 12% versus 13.7% [19].

All students attending the participating schools were provided with an invitation to participate. Written informed consent was obtained from parents and children provided written assent. Data were collected from two cohorts of children. Data collection for cohort one occurred during the 2014–2015 school year (November–May) and cohort two during the 2015–2016 school year (November–May). The proportion of potential children recruited was 72.3% for cohort 1 and 73.3% for cohort two. Children were included in this study if there were complete motor skills and recreation and leisure participation data. Participants were 400 grade 4 children (51% female; Mean age = 9 years 6 months).

Motor proficiency was assessed using the Test of Gross Motor Development-Second Edition (TGMD-2) [20]. The TGMD-2 is a 12-item, norm- and criterion-referenced measure of fundamental motor skills. The test has two subtests: (1) locomotor skills (run, gallop, slide, hop, jump and leap); and (2) object control skills (dribble, catch, strike, throw, roll and kick). Each item on the subtest is assessed individually using a dichotomous rubric. Every time a child performs a skill component of each of the 48 TGMD-2 criteria correctly (e.g., when a child jumps, his or her “preparatory movement includes flexion of both knees with arms extended behind body”), he or she is awarded a ‘1’. If the criterion is not met, the child receives a ‘0’. The score range of each subscale if 0–48. The TGMD-2 is validated for children 3 years of age up to 11 years of age. Test-retest reliability is reported at 0.94 (locomotor) and 0.96 (object control) for 6–8 year olds, and 0.86 (locomotor) and 0.84 (object control) for 9–10 year olds. Content validity was established by selecting skills that were frequently taught in pre-school and early elementary school grades and were representative of the gross motor domain. The goodness-of-fit index for the subtests (required to be 0.90 or higher) was reported at 0.96. Criterion-prediction validity was established through moderate to strong partial correlations (locomotor = 0.63; object control = 0.41) between the TGMD-2 and the Basic Motor Generalizations subtest of the *Comprehensive Scales of Student Abilities*.

Activity participation was evaluated using the Children’s Assessment of Participation and Enjoyment (CAPE) [21]. The CAPE measures participation in 55 recreation and leisure activities in which children have voluntarily participated, outside mandated school hours, in the previous four months. The CAPE is sub-divided into nine activity categories, two of which are Organized Sports and Active Physical Recreation [21]. As per the CAPE manual, Organized Sports are ‘Formal’ activities and are defined as structured activities that have set practice times, games, and/or coaching sessions but may or may not be competitive [21]. Active Physical Recreation is ‘Informal’ and includes activities that require physical exertion [21]. The CAPE assesses five dimensions of participation: Diversity (the number of activities done), Intensity (how often an activity is done), With Whom, Where, and Enjoyment (of participating in activities). In this study only Diversity and Intensity are included in the analysis. Diversity is recorded dichotomously. If a child has participated in an activity in the four months prior to administration of the CAPE, they receive a ‘1’ and are asked to provide responses for the remaining dimensions of that activity. If a child has not participated, they receive a ‘0’ and move on to the next activity. The Intensity scores represent how often a child participated in an activity the indicated they did i.e. ‘1’ on the Diversity dimension. Scores ranged from 1 (everyday) to 7 (once in the previous 4 months). These scores (i.e., 1–7) were summed for each category of participation and divided by the total number of items for that category [21]. If a child did not participate in an activity, a ‘0’ was recorded for Intensity of that particular activity. 

The CAPE is a valid and reliable measurement tool designed for use with individuals aged 6–21 years [21]. Test-retest reliability was established by 427 participants who completed the same test twice under similar conditions. Test-retest correlations for Diversity ranged from 0.67 to 0.77 and 0.72 to 0.81 for Intensity [21]. Cronbach’s coefficient alpha was used to determine internal consistency and was calculated for the five activity types. Test content validity was established through a thorough literature review on participation, expert review, and pilot work [21].

The motor skills assessment was completed in the school gymnasium during physical education classes. All children participated during the data collection sessions, however only those with consent to participate were recorded using digital video (Sony Handycam hdr-cx240). Research assistants were trained by the project coordinator and senior investigator to administer the TGMD-2 according to the procedure outlined in the TGMD-2 Examiner’s Manual [20]. Prior to data collection, research assistants completed a practical orientation to each TGMD-2 station. The project coordinator and senior investigator were present at all data collection sessions to ensure the TGMD-2 was administered correctly. Classes were divided into groups of approximately seven children and rotated through testing stations in the school gymnasium. Each station had one research assistant present to videotape the participants, and another to demonstrate the skills and facilitate the running of the station. Each station contained a different combination of locomotor and object controls skills. Each participant was familiarized with the TGMD-2 as per the Examiner’s Manual and completed the each station once. After testing was completed, the digital video data was downloaded on a secure server at the University of Victoria and scored by the primary investigator who had been trained in scoring procedures consistent with the TGMD-2 manual [20]. Digital video scoring of the TGMD-2 has been used in previous studies [11,13]. In this study, video recording allowed for data collection of many participants during the short duration of physical education classes. 

A team of trained research assistants individually administered the CAPE to each participant once according to the protocols outlined in the CAPE manual [21]. Prior to data collection, research assistants practiced administering the CAPE to one another under the instruction of the project coordinator and senior investigator. The CAPE is administered in a quiet setting (e.g., school library) using a picture book that corresponds to the CAPE questionnaire with drawings for each of the 55 activities. Prior to beginning the questionnaire, participants were reminded that they were only to respond with activities they had participated in during the previous four months when they were not at school. They were also reminded there were no ‘right or wrong answers’.

Descriptive statistics and chi-squared analyses were used to examine activity patterns and whether these patterns differed by gender. Pearson product-moment correlation coefficients were used to evaluate the relationship between fundamental motor skill proficiency (locomotor and object control skills) and Diversity and Intensity of both the Organized Sports and Active Physical Recreation activities. A multivariate analysis of variance (MANOVA) with age as a covariate and gender as a factor was used to examine gender-based differences in motor skill proficiency and Intensity of participation in 17 activities. All statistics were computed using SPSS version 23. The Percent Agreement Method ([Number of Agreements/(Number of Agreements + Disagreements) × 100]) [22] was used to calculate inter-observer reliability for the TGMD-2. Percent agreement ranged from 73.0% to 94.0%, with a mean of 84.1%.

## 3. Results

On average, 44.1% of boys and 48.5% of girls participated in Active Physical Recreation, while 33.8% of boys and 37.8% of girls participated in Organized Sports. The prevalence of participation in each of the Organized Sports and Active Physical Recreation activities is reported in Table 1. Chi-squared analysis (see Table 1) revealed that there were multiple significant gender-based differences in activity participation. Of the 17 activities that were compared, nine were significantly different, the majority favoring girls, with the exception of team and snow sports, which favored boys. Frequency statistics revealed that, of the 400 participants, every child participated in at least one of the Organized Sports or Active Physical Recreation activities. On average, boys participated in 6 of the 11 Active Physical Recreation Activities (24.6%) and 2 of the 6 Organized Sports (34.4%). Girls participated in 5/11 Active Physical Recreation Activities (21.0%) and 2/6 Organized Sports (26.8%).

Descriptive statistics for the Intensity of participation are presented in Table 2. Intensity scores were normally distributed for Organized Sports and Active Physical Recreation with no significant skewness or kurtosis for boys or girls. The MANOVA revealed an overall effect (*F*(19, 355) = 6.01, *p* < 0.001; Wilks’ Lambda = 0.754) and a main effect of gender (*F*(19, 355) = 14.31, *p* < 0.001, Wilks’ Lambda = 0.566). Follow-up univariate tests revealed gender-based differences in Intensity of 11 activities (see Table 2); the nine that differed in terms of participation rate and two additional activities, ‘Walk/Hike’ and ‘Riding Bikes’. Of the 11 activities that differed in Intensity by gender, 7 of these differences favored girls, and 5 favored boys.

Table 2 also shows the mean raw scores for object control skills and locomotor skills. There was no significant gender-based difference in locomotor skill proficiency, but boys had significantly higher object control skills. None of the participants in this study achieved the maximum score (i.e., 48) on the locomotor subscale. One male participant achieved the maximum score (i.e., 48) on the object control subscale. A calculation of the percentage of maximum possible score ([(observed score − minimum score)/(maximum score − minimum score)] × 100) [23] revealed that, on average, boys scored 68.0% on the locomotor subscale and 74.2% on the object control subscale. Girls scored an average of 69.4% on the locomotor subscale and 59.5% on the object control subscale.

Pearson product-moment correlation coefficients (see Table 3) were used to examine the relationships between specific activities and fundamental motor skill proficiency (locomotor and object control separately) of boys and girls who participated in the activities. Girls’ locomotor skills were correlated with intensity of participation in gymnastics and boys’ locomotor and object control proficiency were correlated with intensity of participation in team sports. Negative correlations were found between boys’ object control skills and snow sports, as well as between girls’ locomotor skills and water sports.

## 4. Discussion

The vast majority of children in this study had engaged in physical activities in the previous 4 months. This rate of participation is consistent with the high levels of accelerometer measured physical activity among children in this community [15]. The highest prevalence of participation for both boys and girls was in playing informal games (e.g., soccer in the park) followed by going for a walk or hike. There was also high rate of participation (more than 50% of boys and girls) in swimming, playing on equipment, riding bikes/skateboarding, and team sports. This study took place in a community with a higher than average socio-economic status, and therefore children in this community may be afforded more opportunities to participate in activities that have cost associated with (e.g., team sports). Additionally, the temperate climate in Victoria allows for participation of many outdoor activities (e.g., riding bikes, playing on equipment) year-round.

Although boys and girls had the greatest prevalence of participation in the same six activities, there was some variation in order between boys and girls (e.g., girls’ third highest activity was swimming whereas boys’ was riding bikes). Of these top six activities, significantly more girls participated in five of the activities (playing games, walking/hiking, swimming, playing on equipment, and riding bikes), all of which are categorized as informal, while more boys participated in team sports, which is categorized as a formal activity.

Not only did more boys participate in team sports, but among the boys and girls who participated in team sports, boys participated more often than girls. This may mean that boys in this study would experience higher rates of instruction, coaching, and practice time in a formal, competitive or recreational, team sport environment. Participating in team sports has many physical, psychological, and social benefits [24,25]. Physically, children who participate in team sports demonstrate improved cardiovascular fitness and enhanced motor coordination [25]. Psychologically, children who participate in team sports can also develop positive self-esteem [26]. Socially, children who participate in organized physical activities (e.g., team sports) can learn emotional control, conflict resolution, and skills for developing relationships with coaches, friends, and teammates [5]. It is important that more approaches are taken to encourage girls to participate in team sports if it is something they desire to do. If it is not, it is important to find out what it is that will attract girls to participate in activities where they can derive similar benefits.

As well as gender-based differences in types of participation, there were differences in motor skill proficiency. While the boys’ and girls’ locomotor skills levels did not differ, boys demonstrated higher object control skill proficiency than girls. These results are consistent with previous literature [13,27,28,29]. This is perhaps not surprising as boys in this study were participating more frequently in activities that involved ball skills (e.g., team sports, and informal games such as soccer in the park or road hockey). This difference may be particularly important since it is object control skills, rather than locomotor skills, that are significantly associated with higher physical activity levels [1,14]. In addition, Barnett and colleagues’ [14] found that object control skill proficiency in childhood was predictive of participation in moderate-to-vigorous and organized physical activity when they followed-up with the same children in adolescence.

It is unclear why boys and girls at this age have different motor abilities. Physiologically, there is no reason for girls to have less developed object control skills than boys before puberty [30], and the differences are very likely environmental. A qualitative study of physical education participation with grade 5 girls revealed that girls may be insecure about their performance of motor skills in front of their peers [31]. It was found that some participants felt embarrassed or nervous when they were asked to perform a skill in front of their peers, and that one participant would attempt to miss physical education when she knew they would be doing a skill in which she didn’t feel competent [31]. As the sample of grade 4 students in the current study is similar in age to a grade 5 population, these feelings of insecurity may suggest why girls in the current study do not participate as frequently in certain activities such as team sports. Other research has shown that even when girls do participate in territory-invasion games, they may behave as ‘spectator-players’ rather than being fully engaged [32]. Future research should investigate why boys and girls have different levels or participation in activities and motor proficiency. It will be important to determine if girls are not participating as frequently because they do not feel they have the skills, or simply because they do not prefer the activities that boys participate in. If girls are not participating in these types of activities because they lack, or perceive they lack, motor skill proficiency, appropriate interventions can be developed. This is not to say, however, that girls are not participating in many activities. As mentioned, more girls in this study are participating in informal physical activities than boys and therefore, likely to be accruing the associated physical and psychosocial benefits [33,34]. However, their choices should not be limited by gaps in their movement repertoire or feelings of unskillfulness.

Surprisingly, there were fewer significant correlations between motor skill proficiency and intensity of activity participation than anticipated. There was a negative correlation between girls’ locomotor skills and water sports, as well as a negative correlation between boys’ object control skills and snow sports. While these relationships were modest, it is important to note that the intensity of participation of water and snow sports was quite low (i.e., 1 time in the previous 4 months). It is possible that participation in these activities is low with this population as the city of Victoria is not necessarily conducive to snow sports or water sports. Additionally, however, the skills assessed in the TGMD-2 do not necessarily capture the skills required to participate in water or snow sports, as there are no aquatic or balance components included. Therefore, the relationships between water and snow sports and motor skill proficiency must be looked at discerningly. To date, there have been no published studies using objective measures of validity for the CAPE which is a limitation to this study. Future research would benefit from the addition of objective measures provided they were collected to match the time frame of the CAPE (e.g., collected during the four months prior to CAPE administration).

Gymnastics was the only activity positively correlated with girls’ motor skills, specifically their locomotor skills. This is suggestive of the importance of the children’s experiences in developing motor skills, but also of the skills captured by the TGMD-2. Locomotions, springing, and landing are 3 of the 6 foundational movement patterns of educational gymnastics [35]. These movement patterns would foster the development of skills within the TGMD-2 such as leaping, jumping, hopping, and galloping. As such, it is surprising that there was no significant correlation between dancing and locomotor skills, as dancing can include many similar movement patterns. This may be the result of children in this study being asked about their participation in informal dancing as opposed to formal dance lessons that may include more locomotor skill instruction and practice. It also builds on previous research using the same tools in the same schools with younger children [9,12]. Neither of those studies found significant associations between girls’ locomotor skills and intensity of participation in organized sports in kindergarten or grade 2. The significant relationship between gymnastics participation and locomotor skills among the girls in this study is consistent with developmental theory that the relationship between motor skills and physical activity should strengthen as children transition from early childhood to middle childhood [30]. Similarly, boys’ locomotor and object control skills were positively correlated with participation in team sports. Team sports such as soccer, basketball, and hockey require the manipulation of objects as well as moving in multiple ways. It is perhaps not surprising that boys with higher rates of participation have more developed motor skills. However, it is also possible that boys with better motor skills are choosing and persisting with team sports and the girls with better locomotor skills are doing the same with gymnastics. There is nothing wrong with children becoming more skillful through their participation, but this study has not examined whether all children feel they are able to participate. Again, prior research by our team with kindergarten and grade 2 children in the same schools using both the CAPE and TGMD-2 demonstrated significant and positive relationships between boys’ object control skills and participation in organized sports [13,16], but not for locomotor skills. For the boys in grade 4 in this study, the relationship with organized sports was also significant for locomotor skill level. This expansion of relationships between participation and motor skills is consistent with Stodden and colleagues’ thoughts that in middle childhood “… higher levels of motor skill competence will offer a greater motor repertoire to engage in various physical activities, sports, and games” [30] (p. 295), and that moderately and highly skilled children may begin to self-select into physical activities [30].

## 5. Conclusions

Boys and girls in this study showed similar prevalence of participation in a number of physical activities. However, more girls participated in informal physical activities than did boys, and more boys participated in formal team sports. Additionally, boys participated more frequently in activities that included object control skills (e.g., playing games and team sports). Results revealed similar locomotor proficiency for boys and girls, but different object control proficiency. This may be because spending time in object control dominated activities provides opportunity for practice and skill improvement. Unexpectedly, there were few associations with physical activities and motor skill levels. Future research should investigate the reasons behind why boys and girls choose to participate in certain physical activities and not others in order to better understand participation trends.

## Figures and Tables

**Table 1:** Prevalence of participation in sports and active recreation activities as measured by the Children’s Assessment of Participation and Enjoyment (CAPE).

Item	All (%)	Boys (%)	Girls (%)	*p*
**Organized sports**				
Swimming	77.3	70.3	88.2	0.001
Gymnastics	23.0	12.0	35.4	<0.001
Horse riding	9.5	4.1	15.4	<0.001
Martial arts	17.8	24.1	12.3	0.001
Track & Field	32.0	29.2	36.4	0.247
Team sports	55.5	63.1	50.8	0.003
**Active physical recreation**				
Walk/hike	82.3	79.0	89.7	0.094
Playing on equipment	76.3	69.7	86.7	0.003
Dancing	39.3	21.5	59.0	<0.001
Bike riding/skateboard	76.0	75.9	76.1	0.963
Water sports (excluding swimming)	19.5	19.0	21.0	0.796
Snow sports	20.3	24.6	16.9	0.034
Games (e.g., soccer at the park)	85.3	83.6	91.3	0.361
Gardening	28.8	22.1	36.9	0.004
Fishing	14.8	17.4	12.8	0.140
Individual physical activities	38.0	42.6	33.7	0.067
Non-team sports	29.8	29.7	31.3	0.998

**Table 2:** Mean scores for motor skills and intensity of activity participation.

Variable (Score Range)	Boys (*n* = 195)		Girls (*n* = 205)		*p*
	Mean	SD	Mean	SD	
**Motor Proficiency**					
Locomotor skills raw score (0–48)	32.6	4.5	33.3	4.5	0.077
Object control skills raw score (0–48)	35.6	5.5	28.6	5.8	<0.001
**Organized Sports (1–7)**					
Swimming	3.2	2.4	3.8	2.1	0.002
Gymnastics	0.6	1.6	1.8	2.6	<0.001
Horse riding	0.1	0.4	0.3	1.1	0.004
Martial arts	1.3	2.4	0.6	1.7	<0.001
Track & Field	1.3	2.3	1.6	2.4	0.340
Team sports	3.6	2.9	2.6	2.9	<0.001
**Active Physical Recreation (1–7)**					
Walk/hike	3.3	2.4	3.9	2.1	0.008
Playing on equipment	3.8	2.9	4.8	2.6	<0.001
Dancing	1.0	2.1	2.9	2.8	<0.001
Bike riding/skateboard	4.0	2.7	3.5	2.5	0.021
Water sports (excluding swimming)	0.7	1.6	0.7	1.7	0.852
Snow sports	0.5	1.1	0.3	0.7	0.014
Games (e.g., soccer at the park)	5.1	2.6	5.0	2.4	0.666
Gardening	0.9	1.9	1.2	1.9	0.042
Fishing	0.4	1.0	0.3	0.8	0.340
Individual physical activities	2.3	2.9	1.7	2.6	0.071
Non-team sports	1.2	2.1	1.4	2.3	0.599

Notes: Intensity score: 0 = did not participate in the past four months; 1 = one time in the past four months; 2 = two times in the past four months; 3 = one time a month; 4 = two or three times a months; 5 = one time a week; 6 = two or three times a week; and 7 = one or more times per day).

**Table 3:** Relationships between intensity of activity participation and motor skill proficiency.

Activity	Boys		Girls	
	Locomotor	Object Control	Locomotor	Object Control
**Organized Sports**				
Swimming	−0.136	0.055	−0.056	−0.092
Gymnastics	−0.159	0.052	0.333 **	−0.039
Horse riding	−0.551	−0.361	0.088	0.011
Martial arts	−0.076	−0.177	0.006	0.134
Track & Field	0.003	0.008	0.077	0.223
Team sports	0.210 *	0.225 *	−0.023	0.117
**Active Physical Recreation**				
Walk/hike	−0.102	0.086	−0.010	−0.032
Playing on equipment	−0.010	0.120	0.043	0.066
Dancing	−0.085	0.159	0.122	−0.031
Bike riding/skateboard	−0.085	0.108	0.067	−0.038
Water sports (excluding swimming)	−0.042	−0.222	−0.353 *	−0.027
Snow sports	−0.087	−0.357 *	−0.155	−0.207
Games (e.g., informal soccer at the park)	−0.005	0.074	−0.067	0.016
Gardening	0.081	0.172	−0.130	0.003
Fishing	−0.033	0.211	−0.296	−0.229
Individual physical activities	−0.035	0.146	−0.017	0.023
Non-team sports	−0.203	0.012	−0.051	−0.144

Notes: * Correlation is significant at *p* < 0.05, ** Correlation is significant at *p* < 0.01.

## References

[1] Wrotniak B.H., Epstein L.H., Dorn J.M., Jones K.E., Kondilis V.A. (2006). The relationship between motor proficiency and physical activity in children. Pediatrics.

[2] Barnett L.M., Morgan P.J., van Beurden E., Beard J.R. (2008). Perceived sports competence mediates the relationship between childhood motor skill proficiency and adolescent physical activity and fitness: A longitudinal assessment. Int. J. Behav. Nutr. Phys. Act..

[3] McCurdy L.E., Winterbottom K.E., Mehta S.S., Roberts J.R. (2010). Using nature and outdoor activity to improve children’s health. Curr. Probl. Pediatr. Adolesc. Health Care.

[4] Janz K.F., Thomas D.Q., Ford M.A., Williams S.M. (2015). Top 10 research questions related to physical activity and bone health in children and adolescents. Res. Q. Exerc. Sport.

[5] Eime M.R., Young J.A., Harvey J.T., Charity M.J., Payne W.R. (2013). A systematic review of the psychological and social benefits of participation in sport for children and adolescents: Informing development of a conceptual model of health through sport. Int. J. Behav. Nutr. Phys. Act..

[6] Cowart B.L., Saylor C.F., Dingle A., Mainor M. (2004). Social skills and recreational preferences of children with and without disabilities. N. Am. J. Psychol..

[7] Brown T., O’Keefe S., Stagnitti K. (2011). Activity preferences and participation of school-age children living in urban and rural environments. Occup. Ther. Health Care.

[8] Findlay L.C., Garner R.E., Kohen D.E. (2010). Patterns of children’s participation in unorganized physical activity. Res. Q. Exerc. Sport.

[9] King G., Law M., King S., Hurley P., Hanna S., Kertoy M., Rosenbaum P. (2007). Measuring children’s participation in recreation and leisure activities: Construct validation of the cape and pac. Child: Care Health Dev..

[10] Temple V.A., Foley J.T. (2016). A peek at the developmental validity of the test of gross motor development—3. J. Motor Learn. Dev..

[11] LeGear M., Greyling L., Sloan E., Bell R., Williams B., Naylor P., Temple V.A. (2012). A window of opportunity? Motor skills and perceptions of competence of children in kindergarten. Int. J. Behav. Nutr. Phys. Act..

[12] Payne G.V., Isaacs L.D. (2012). Human Motor Development: A Lifespan Approach.

[13] Temple V.A., Crane J.R., Brown A., Williams B., Bell R.I. (2016). Recreational activities and motor skills of children in kindergarten. Phys. Educ. Sport Pedagog..

[14] Barnett L.M., van Beurden E., Morgan P.J., Brooks L.O., Beard J.R. (2009). Childhood motor skill proficiency as a predictor of adolescent physical activity. J. Adolesc. Health.

[15] Crane J.R., Naylor P.J., Cook R., Temple V.A. (2015). Do perceptions of competence mediate the relationship between fundamental motor skill proficiency and physical activity levels of children in kindergarten?. J. Phys. Act. Health.

[16] Mirjafari E. (2015). The Relationship between Motor Skills, Perceptions of Competence, and Participation in Active Recreation and Physical Activities. Master’s Thesis.

[17] Clark J.E., Metcalfe J.S. (2002). The mountain of motor development. Motor Dev. Res. Rev..

[18] Statistics Canada (2016). Median Total Income, by Family Type, by Census Metropolitan Area (All Census Families).

[19] Kershaw P., Irwin L., Trafford K., Hertzman C. (2005). The British Columbia Atlas of Child Development.

[20] Ulrich D.A. (2000). Test of Gross Motor Development (tgmd-2).

[21] King G., Law M., King S., Hurley P., Rosenbaum P., Hanna S., Kertoy M., Young N. (2004). Children’s Assessment of Participation and Enjoyment & Preferences for Activities for Children.

[22] Baumgartner T.A., Strong C.H., Hensley L.D. (2002). Conducting and Reading Research in Health and Human Performance.

[23] Cohen P., Cohen J., Aiken L.S., West S.G. (1999). The problem of units and the circumstance for pomp. Multivar. Behav. Res..

[24] Krustrup P., Dvorak J., Junge A., Bangsbo J. (2010). Executive summary: The health and fitness benefits of regular participation in small-sided football games. Scand. J. Med. Sci. Sports.

[25] Vandendriessche J.B., Vandorpe B.F.R., Vaeyens R. (2012). Variation in sport participation, fitness and motor coordination with socioeconomic status among flemish children. Pediatr. Exerc. Sci..

[26] Findlay L.C., Coplan J. (2008). Come out and play: Shyness in childhood and the benefits of organized sports participation. Can. J. Behav. Sci..

[27] Barnett L.M., Ridgers N.D., Salmon J. (2015). Associations between young children’s perceived and actual ball skill competence and physical activity. J. Sci. Med. Sport.

[28] Barnett L.M., van Beurden E., Morgan P., Brooks L.O., Beard J.R. (2010). Gender differences in motor skill proficiency from childhood to adolescence. Res. Q. Exerc. Sport.

[29] Crane J.R., Temple V.A. (2016). An Examination of the Relationships between Fundamental Motor Skills, Perceived Physical Competence, and Physical Activity Levels during the Primary Years. Ph.D. Thesis.

[30] Gabbard C.P. (2012). Lifelong Motor Development.

[31] Gibbons S. (2008). Female student perceptions of grade 5 physical education. Phys. Health Educ. J..

[32] Gutierrez D., García-López L.M. (2012). Gender differences in game behaviour in invasion games. Phys. Educ. Sport Pedagog..

[33] Coşoveanu S., Bulucea D. (2010). Study on the relationship between lifestyle and obesity in kindergarden and primary school children. Acta Medica Marisiensis.

[34] Holder M.D., Coleman B., Sehn Z.L. (2009). The contribution of active and passive leisure to children’s well-being. J. Health Psychol..

[35] Thompson K., Robinson D. (2016). Gymnastics for every student. Phys. Health Educ. J..

